# Nitazoxanide, an Antiprotozoal Drug, Reduces Bone Loss in Ovariectomized Mice by Inhibition of RANKL-Induced Osteoclastogenesis

**DOI:** 10.3389/fphar.2021.781640

**Published:** 2021-12-09

**Authors:** Chang-hong Li, Zi-rui Lü, Zhen-da Zhao, Xin-yu Wang, Hui-jie Leng, Yan Niu, Mo-pei Wang

**Affiliations:** ^1^ Department of Rheumatology and Immunology, Peking University Third Hospital, Beijing, China; ^2^ Osteoporosis and Bone Metabolic Diseases Center, Peking University Third Hospital, Beijing, China; ^3^ Department of Medicinal Chemistry, School of Pharmaceutical Sciences, Peking University Health Science Center, Beijing, China; ^4^ Department of Orthopaedics, Peking University Third Hospital, Beijing, China; ^5^ Beijing Key Laboratory of Spinal Disease Research, Beijing, China; ^6^ Department of Tumor Chemotherapy and Radiation Sickness, Peking University Third Hospital, Beijing, China

**Keywords:** nitazoxanide, stat3, osteoclastogenesis, Nfatc1, osteoporosis

## Abstract

Nitazoxanide (NTZ) is an FDA-approved anti-parasitic drug with broad-spectrum anti-infective, anti-inflammatory, and antineoplastic potential. However, its regulatory effects on osteoclastogenesis and the underlying mechanisms remain unclear. The present study found that NTZ potently inhibited osteoclast formation at the early stage of receptor activator of NF-κB ligand-induced osteoclastogenesis in a concentration-dependent manner at a non-growth inhibitory concentration. NTZ suppressed actin ring formation and decreased osteoclast marker gene expression, including TRAP, MMP9, and cathepsin K. NTZ significantly impaired the bone resorption activity of osteoclasts. *In vivo*, ovariectomized mice were treated with 50, 100 and 200 mg/kg/d NTZ for 3 months. NTZ (100 mg/kg/d) administration markedly reduced ovariectomy-induced bone loss by suppressing osteoclast activity. Mechanistically, osteoclastogenesis blockade elicited by NTZ resulted from inhibition of STAT3 phosphorylation, and reduction of the Ca^2+^ fluorescence intensity and NFATc1 expression. NTZ weakened the binding between STAT3 and the NFATc1 promoter region. Furthermore, enforced NFATc1 expression partly rescued the impaired osteoclast differentiation in NTZ-treated RAW264.7 cells. In summary, NTZ could inhibit osteoclastogenesis and bone loss through modulation of the receptor activator of NF-κB ligand-induced STAT3-NFATc1 signaling pathway, which might be a potential alternative treatment regimen against bone destruction-related diseases including osteoporosis.

## Introduction

Excess osteoclastic activity disrupts the balance between bone-forming osteoblasts and bone resorbing osteoclasts in bone remodeling and causes osteolytic diseases, including osteoporosis (OP) (Boyle et al., 2003). As the aging population continues to increase, OP has become a frequent and common disease that seriously affects the quality of life (Hernlund et al., 2013; Compston et al., 2019). It is estimated that there are billions of OP patients globally, particularly among the elderly (Black and Rosen, 2016; Brown, 2017). Fortunately, several forms of effective treatments are available in accordance with the endocrine society clinical practice guideline published in the Journal of Clinical Endocrinology & Metabolism in 2019 (Eastell et al., 2019). However, patients that use bisphosphonates or denosumab, which are the firstline anti-resorptive therapies, remain at risk of adverse events, including atypical femoral fracture and osteonecrosis of the jaw (Van den Wyngaert et al., 2011; Black et al., 2019). Therefore, it is necessary to develop new anti-osteoporosis drugs with clear molecular mechanisms and improve the treatment status of osteolytic diseases.

Osteoclasts, a kind of multinucleated giant cells derived from the monocyte/macrophage lineage, are responsible for bone loss. Macrophage colony-stimulating factor (M-CSF) and receptor activator of NF-κB ligand (RANKL) are two critical extracellular factors for osteoclastogenesis (Boyle et al., 2003). M-CSF mainly facilitates osteoclast precursors proliferation and survival (Tanaka et al., 1993), while RANKL supports osteoclast differentiation and activation (Jimi et al., 1999). Binding of RANKL to receptor RANK initiates a series of downstream signal cascades, including the NF-κB, MAPK, and PI3K/AKT pathways, and thereby triggers osteoclast precursors to differentiate into osteoclasts by inducing osteoclast associated genes expression, including tartrate-resistant acid phosphatase (TRAP), cathepsin K, c-Fos, and NFATc1 (Park et al., 2017). A variety of small molecule compounds that target those downstream pathways have osteoprotective effects (Xie et al., 2018; Hsu et al., 2019; Zhao et al., 2019), which further confirmed that inhibiting the downstream signaling pathways activated by RANKL may be beneficial in the treatment of bone-destructive diseases. Additionally, our previous study (Li et al., 2018) and the recent study by Yang et al., 2019) suggested that the signal transducer and activator of transcription 3 (STAT3) signaling pathway also plays a crucial role in RANKL-induced osteoclastogenesis. Therefore, we hypothesized that targeting the RANKL-activated STAT3 signaling pathway may efficaciously suppress osteoclast formation, which can be used as a potential treatment against osteolytic bone diseases.

Nitazoxanide (NTZ, Supplementary Figure S1), a thiazolide antiparasitic agent, is approved by the U.S. Food and Drug Administration (FDA) for the treatment of *Giardia intestinalis* and *Cryptosporidium parvum* infection in non-immunodeficient children and adults (White, 2004). In recent years, many independent studies have demonstrated that NTZ has a broad spectrum of pharmacological actions against infectious and neoplastic diseases (Di Santo and Ehrisman, 2014; Rossignol, 2014; Shakya et al., 2018). Mechanistically, the signaling pathways which NTZ can regulate including Wnt/β-catenin pathway (Qu et al., 2018), c-Myc (Fan-Minogue et al., 2013), and mTORC1 (Lam et al., 2012). Our recent study identified NTZ as an inhibitor of STAT3 that significantly inhibits STAT3 pathway transactivation in cancer cells (Lü et al., 2021). The present study hypothesized that NTZ might have a potential inhibitory effect on RANKL-mediated osteoclastogenesis by targeting STAT3 signaling. Whether NTZ prevented osteoclast-associated osteoporosis was investigated both *in vitro* and *in vivo.*

## Materials and Methods

### Reagents and Antibodies

Recombinant murine RANKL and M-CSF were supplied by Peprotech (Rocky Hill, NJ, USA). Phospho-IκBα, IκBα, phospho-JNK, JNK, p38, phospho-p38, ERK, phospho-ERK, STAT3, and phospho-STAT3 antibodies were purchased from Cell Signaling Technology (Beverly, MA, USA). Antibodies against NFATc1, c-Fos and β-actin were obtained from Santa Cruz Biotechnology (Santa Cruz, CA, USA). NTZ with purity greater than 99% was purchased from Selleck Chemicals (Shanghai, China).

### Cell Culture, Osteoclast and Actin-Ring Formation

Primary bone marrow monocytes (BMMs) were obtained from the femur and tibia of 6-week-old female C57BL/6J mice as previously described (Li et al., 2021). Briefly, the cells were isolated by flushing the femoral and tibial bone marrow with minimum essential medium-α (Gibco, NY, USA) supplemented with 10% fetal bovine serum (FBS) and penicillin-streptomycin (PS). Then osteoclast precursor cells (preosteoclasts) were induced under the stimulation with M-CSF (30 ng/ml) for 2 days. RAW264.7, provided by Peking Union Medical College (Beijing, China), is a mononuclear macrophage cell line and cultured in high glucose Dulbecco’s Modified Eagle Medium supplemented with 10% FBS and PS. For osteoclast differentiation, preosteoclasts were cultured in the osteoclast induction medium with the addition of M-CSF (10 ng/ml), RANKL (50 ng/ml), and different concentrations of NTZ (0, 10, 20 and 40 µM) for 7 days. Before osteoclast maturation, the medium was changed every 3 days. Then, osteoclasts were fixed with 4% paraformaldehyde and stained by tartrate-resistant acid phosphatase (TRAP) activity using leukocyte acid phosphatase kit (Sigma-Aldrich, 387A-1KT) according to the manufacturer’s instructions. TRAP positive cells with three or more nuclei were counted as osteoclasts. As for actin-ring formation, mature osteoclasts were preincubated with FITC-phalloidin for 1 h, and then stained with DAPI for 3 min.

### Cell Viability Assay

CellTiter 96^®^ Aqueous One Solution Reagent (Promega, USA) was used to determine the cytotoxic effects of NTZ on BMMs according to the manufacturer’s protocol. Bone marrow monocytes at a density of 1.5×10^4^ cells/well were cultured in a 96-well plate with M-CSF (30 ng/ml) stimulation for 48 h. Then cells were treated in the presence of different concentrations of NTZ (0, 10, 20, 40, 80 and 100 µM) with or without M-CSF (10 ng/ml) for 24 or 48 h (Tantawy et al., 2020). 10 µL of MTS solution was added to each well, and the plate was incubated in the incubator for another 2 h. The 490 nm absorbance was measured using a microplate reader (Thermo Scientific). The cell viability of the blank control well was defined as 100%.

### Bone Resorption Assay

BMMs at a density of 5×10^4^ cells/well were seeded onto bovine bone slices in a 48-well plate with three replicates, and cultured in the presence of M-CSF (30 ng/ml) for 48 h. Then preosteoclasts were further stimulated with M-CSF (30 ng/ml) plus RANKL (50 ng/ml). NTZ (20, 40 µM) was added on day 0 or day 6. After 2 weeks induction, cells around the slices were fixed by 4% paraformaldehyde and stained for TRAP activity. The number of TRAP positive osteoclasts was counted under light microscope. Then, bone slices were removed from the well, fixed with 2.5% glutaraldehyde. Cells on the bone slice surface were clearly swept. 0.5% toluidine blue (Sigma) staining was applied to detect the resorption pits. The erosion area was calculated using ImageJ software (NIH, Bethesda, MD, United States).

### Real-Time Quantitative PCR RNA Expression Analyses

TRIzol reagent (Invitrogen, Carlsbad, CA, USA) was used to isolate total RNA. The mRNA was then converted to complementary DNA (cDNA) with Superscript II reverse transcriptase (Invitrogen). RT-PCR was performed using an iQ5 PCR Detection System (Bio-Rad, Hercules, CA, United States) with 2x SYBR Green Talent qPCR PreMix (TIANGEN, Beijing, China). Relative mRNA level of each target was quantified using the 2^−ΔΔCT^ method and normalized to GAPDH. Supplementary Table S1 provides the details of primers sequences used in this study.

### Western Blotting

Radio-immunoprecipitation assay (RIPA) lysis buffer (Apllygen, Beijing, China) with addition of phosphatase and protease inhibitors was used to extract the total protein. The supernatants were collected after centrifugation at 12,000 *g* for 15 min. The proteins were subjected to SDS-PAGE gels and transferred onto polyvinylidene fluoride (PVDF) membranes (Immobilon-P; Millipore, Billerica, MA, United States). After blocking, the membranes were probed with primary antibodies, including pIκBα, IκBα, pJNK, JNK, p38, p-p38, ERK, pERK, STAT3, pSTAT3, NFATc1, c-Fos (1:1,000) and β-actin (1:5,000) overnight with gentle shaking, followed by incubation with fluorescently labeled secondary antibodies (1:10,000; LI-COR Biosciences, Lincoln, NE). Finally, the bound proteins were visualized using an Odyssey Infrared Imaging System (LI-COR Biosciences, Lincoln, NE, United States). The bands of interest were quantified using ImageJ software.

### Ovariectomized -Induced Bone Loss Model

Twelve-week-old Female C57BL/6 mice were purchased from Charles River (Beijing, China) and subjected to either a sham operation or bilateral ovariectomy to create osteoporosis model (Li et al., 2018). All animal experimental procedures in this study were carried out in accordance with the Guide for the Care and Use Laboratory Animals and the related ethical regulations of Peking University Third Hospital. Because there has not been any *in vivo* study on effective dosage of NTZ in treating bone destruction-related diseases, we referred to a dose (200 mg/kg/bid) commonly used in mice models of protozoan infection that induces maximum growth inhibition without significant side effects (Fan-Minogue et al., 2013). Considering that it often requires long-term dosage in the treatment of osteolytic diseases and the administration of NTZ will last for 3 months in this *in vivo* assay, we adopted 200 mg/kg/d as the high dose and accordingly, set 100 mg/kg/d and 50 mg/kg/d as middle and low dose, respectively. Then mice were randomly divided into five groups: Sham group, OVX group, OVX + 50 mg/kg/d NTZ (Low) group, OVX + 100 mg/kg/d NTZ (Middle) group and OVX + 200 mg/kg/d NTZ (High) group. NTZ was dissolved in 0.5% carboxymethylcellulose (CMC) and mixed by ultrasound. From the third postoperative day, NTZ and CMC were administered intragastrically every day for 3 months. At the end of this experiment, all mice were sacrificed with excess amounts of isoflurane. Dual-energy X-ray absorptiometry (DXA) with a small-animal high-resolution collimator (DiscoveryTM, Hologic Inc., MA, United States) was used to quantify the bone density of the left tibia and femur from each mouse. All the DXA data were analyzed by a professional technician blinded to the experimental procedure. Right tibias of mice were excised, fixed in 4% formaldehyde, decalcified in 10% tetrasodium-EDTA aqueous solution, and embedded in paraffin for hematoxylin and eosin (H&E) and TRAP staining.

### Micro–Computed Tomography Scanning

A high-resolution micro-CT scanner (Inveon, Siemens, IL, United States) was used to analyze the microstructural parameters in the left tibias. Spatial resolution at 13 µm and X-ray energy settings of 80 kV and 500 µA were set for the scanning protocol. The scanned data were imported into the Inveon Research Workplace (Siemens, IL, United States) for image analysis. Eighty continuous slices beginning at 0.1 mm below the growth plate in which condyles were no longer visible were chosen for further qualitative and quantitative analyses. The parameters, including trabecular number (Tb.N), trabecular thickness (Tb.Th), trabecular separation (Tb.Sp) and bone volume/tissue volume ratio (BV/TV), were measured for trabecular analysis among the five groups.

### Three-Point Bending Test

The mechanical properties of the tibias were further analyzed by three-point bending tests. Mechanical loading was applied midway between two supports placed 8 mm apart. When the tibia was positioned, a preload of 0.5 N was applied to the middle of the bone. The center loading plate was moved at a rate of 1 mm/min until bone fracture. The maximum force was determined on the basis of the resulting force data. The slope of the linear portion of the force deflection curve was regarded as stiffness.

### Dual-Luciferase Reporter Assay

The pcDNA3.1-STAT3, NFATc1 promoter-driven pGL3-basic based luciferase reporter plasmid and the corresponding control plasmids were synthesized by SyngenTech (Beijing, China). The pGL3-basic vector contains both firefly and Renilla luciferase reporter genes. The firefly luciferase in the dual-luciferase reporter assay system was used to evaluate promoter activity, and Renilla luciferase was used as a normalization control to correct the relative transfection efficiency. RAW264.7 cells were cultured in a 48-well plate with a density of 3×10^4^ cells/well for 12 h. Then plasmids were transfected into RAW264.7 cells with TurboFect Transfection Reagent (Thermo Scientific) according to the manufacturer’s protocol. At 72 h after transfection, the cell lysis supernatants were used to determine the luciferase activity using the Dual-Luciferase Reporter Assay System according to the manufacturer’s protocol (E1910; Promega, Madison, WI, United States).

### Statistical Analysis

Data are presented as data points with mean ± SD. Statistical analyses were performed using GraphPad Prism 7 (GraphPad Software Inc., San Diego, CA) and SPSS 25 (SPSS Inc.). For the comparison of two groups, unpaired two-tailed Student’s *t* test was used. Multiple comparisons between groups were carried out using one-way ANOVA with Tukey’s post hoc test. Values of *p* < 0.05 were considered statistically significant.

## Results

### NTZ Reduced RANKL-Induced Osteoclastogenesis *in vitro*

**FIGURE 1:**
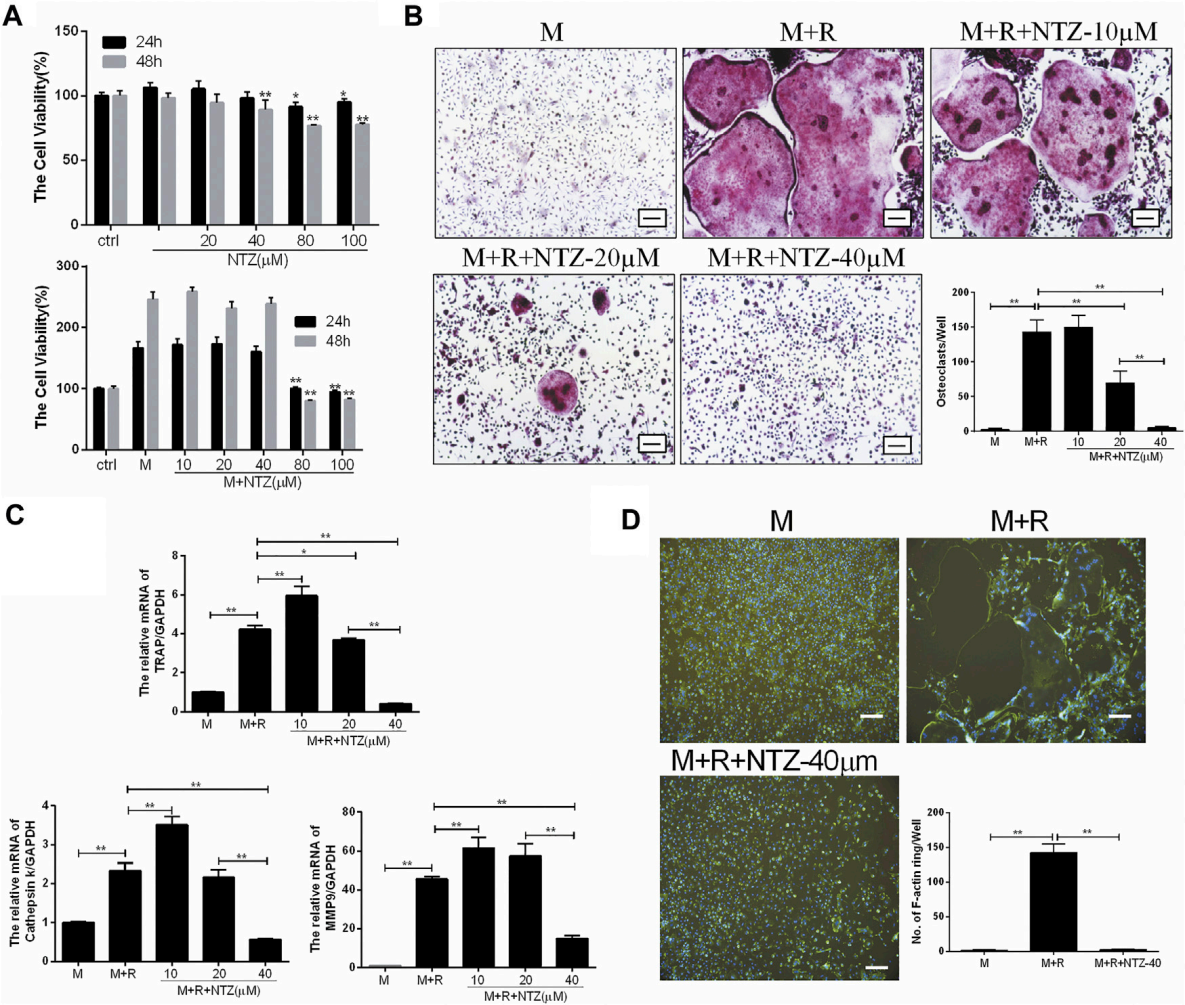
Nitazoxanide inhibited RANKL-mediated osteoclastogenesis *in vitro*. **(A)** Cytoactivation of NTZ-treated BMMs at 24 and 48 h with or without M-CSF stimulation was tested by MTS assays. Values of *p* were compared between experimental group and control group **(top)**, experimental group and M-CSF group **(bottom)**. **(B)** TRAP staining was performed after BMMs treated with various NTZ concentrations, M-CSF (10 ng/ml), and RANKL (50 ng/ml) for 7 days. The number of TRAP-positive multinuclear cells were counted. Scale bar = 100 µm. **(C)** Real-time PCR analyzed the expression of osteoclastic genes including TRAP, Cst K and MMP9. **(D)** BMMs were stimulated with or without RANKL (50 ng/ml) in the presence of M-CSF (10 ng/ml), followed by treatment with or without 40 µM NTZ. Cells were fixed and stained for F-actin. Quantification of the actin ring was by positive F-actin staining. Scale bar = 100 µm. Error bars represent mean ± SD from three independent experiments. ctrl, control; M, M-CSF; NTZ, nitazoxanide; R, RANKL. *p value <0.05, **p value <0.01.

M-CSF-induced BMMs proliferation is the prerequisite for osteoclast differentiation. Firstly, the cytotoxicity of NTZ on primary BMMs was determined for 24 and 48 h. As shown in Figure 1A top, NTZ (≤20 µM) had little effect on BMMs proliferation without M-CSF stimulation at both 24 and 48 h. NTZ at concentrations >40 µM slightly inhibited BMMs proliferation. However, M-CSF addition promoted BMMs proliferation and partly reversed the cytotoxic effect of NTZ on BMMs, particularly at concentrations ≤40 µM (Figure 1A bottom). Accordingly, NTZ concentration of 40 µM was used in subsequent experiments. To further explore the effect of NTZ on RANKL-induced osteoclastogenesis *in vitro*, BMMs were treated with M-CSF and RANKL, as well as NTZ at concentrations of 10, 20, and 40 µM. An apparent suppression of mature osteoclast formation was observed at 40 µM NTZ (Figure 1B). The number of osteoclasts and the detection of their markers further supported this phenomenon (Figures 1B,C). The effect of NTZ on actin ring formation was determined by immunofluorescence assay. The number of actin ring was significantly reduced in the presence of 40 µM NTZ compared with the control group (Figure 1D). Actin ring formation is an important feature of mature osteoclasts during RANKL-mediated osteoclastogenesis, and represents osteoclastic bone resorption (Boyle et al., 2003).

### NTZ Inhibited Osteoclast Differentiation at an Early Stage and Attenuated Bone Resorption *in vitro*

**FIGURE 2:**
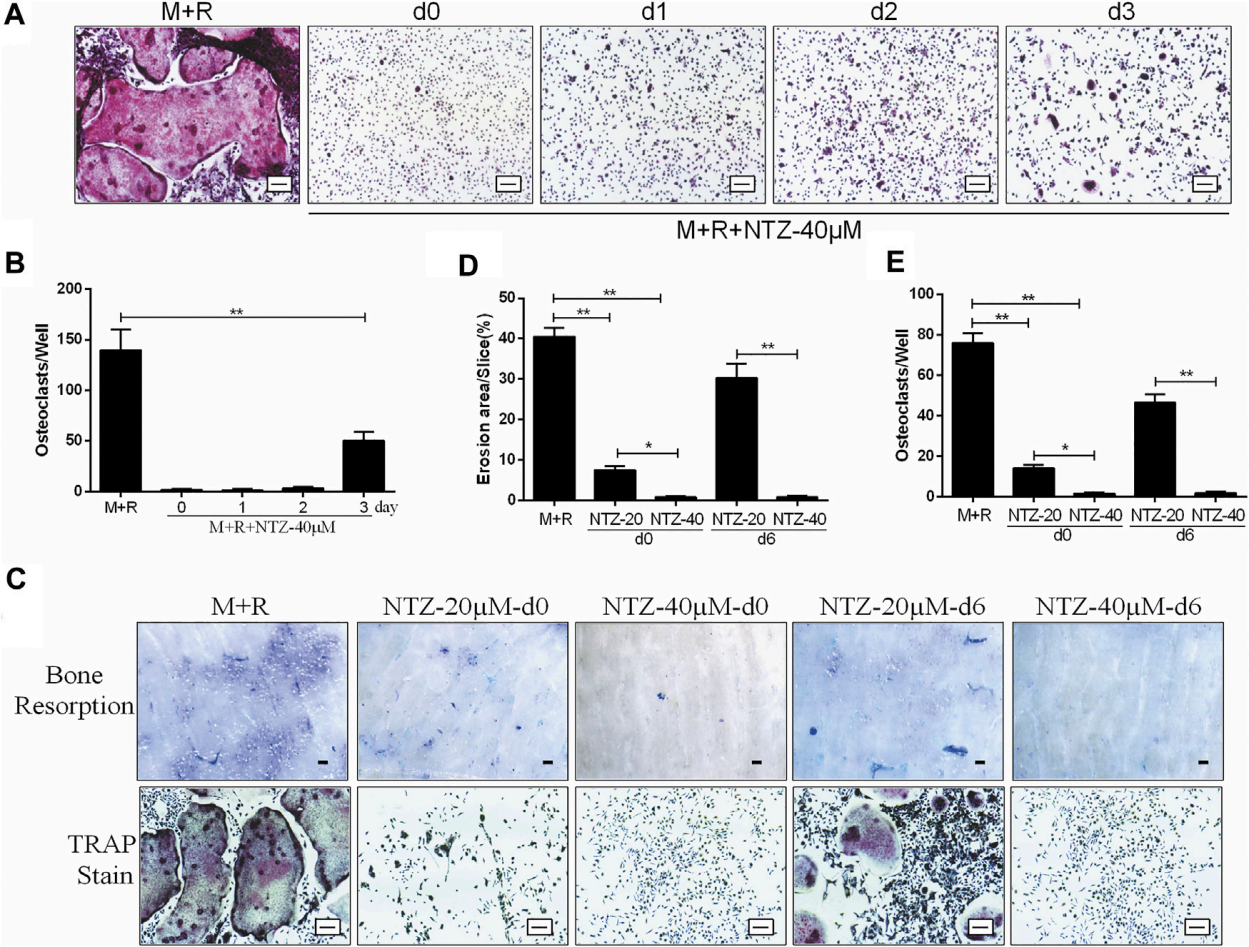
Nitazoxanide inhibited osteoclasts differentiation at an early stage and attenuated osteoclastic bone resorption *in vitro*. **(A)** BMMs were induced to generate osteoclasts in the presence of M- CSF (10 ng/ml) and RANKL (50 ng/ml) for 7 days. NTZ (40 µM) was added to the medium at the indicated times (days 0, 1, 2, or 3). On the day 7, TRAP staining was performed. Scale bar = 100 µm. **(B)** TRAP staining positive multinucleate cells were counted. **(C)** BMMs were seeded onto bone slices in a 48 well plate and treated with M-CSF (10 ng/ml) and RANKL (50 ng/ml), and NTZ (20 or 40 µM) was added at d0 or d6 of the assay for 14 days. Resorption lacuna was stained with toluidine blue **(top)**. Scale bar = 250 µm. The cells around the bone slices were used for TRAP staining **(bottom)**. Scale bar = 100 µm. **(D–E)** The resorption pit areas and number of osteoclasts were quantified. Error bars represent mean ± SD from three independent experiments. d = day; M = M-CSF; NTZ = nitazoxanide; R = RANKL. *p value <0.05, **p value <0.01.

The inhibitive role of NTZ on osteoclastogenesis was adding time dependent based on comparing the osteoclast-induction medium replenished with 40 µM NTZ at different adding time points during the process of osteoclast differentiation. When NTZ was added at an early stage of osteoclast differentiation (days 0–2), osteoclastic formation was strongly inhibited (Figures 2A,B). When NTZ was added on day 3 of osteoclast differentiation, smaller mature osteoclasts were observed, in decreased numbers compared with the M-CSF and RANKL combined treatment group (Figures 2A,B). These results indicated that NTZ exhibited its inhibitive effect on osteoclast formation mainly at the early stage of osteoclast differentiation, and could not reverse the differentiation process once preosteoclasts were committed to the osteoclast lineage.

The effect of NTZ on osteoclastic bone resorption capacity, an important function of the matured osteoclast, is presented in Figures 2C–E. Preosteoclasts were plated on dentin slices in the presence of inductive medium with 20 or 40 µM NTZ for 2 weeks. The area of bone resorption lacunae was decreased when NTZ was added, especially at 40 µM (Figures 2Ci,D), which was directly proved by osteoclast TRAP staining and count (Figures 2Cii, E). When NTZ was added after osteoclasts formation, 40 µM NTZ significantly inhibited bone resorption compared with the 20 µM group (Figures 2Ci,D). This result was also supported by the subsequent osteoclast detection (Figures 2Cii,E). The *in vitro* study showed that NTZ suppressed osteoclast formation mainly at the early stage, and attenuation of bone resorption due to NTZ was concentration-dependent.

### NTZ Ameliorated OVX-Induced Systematic Bone Loss *in vivo*

**FIGURE 3:**
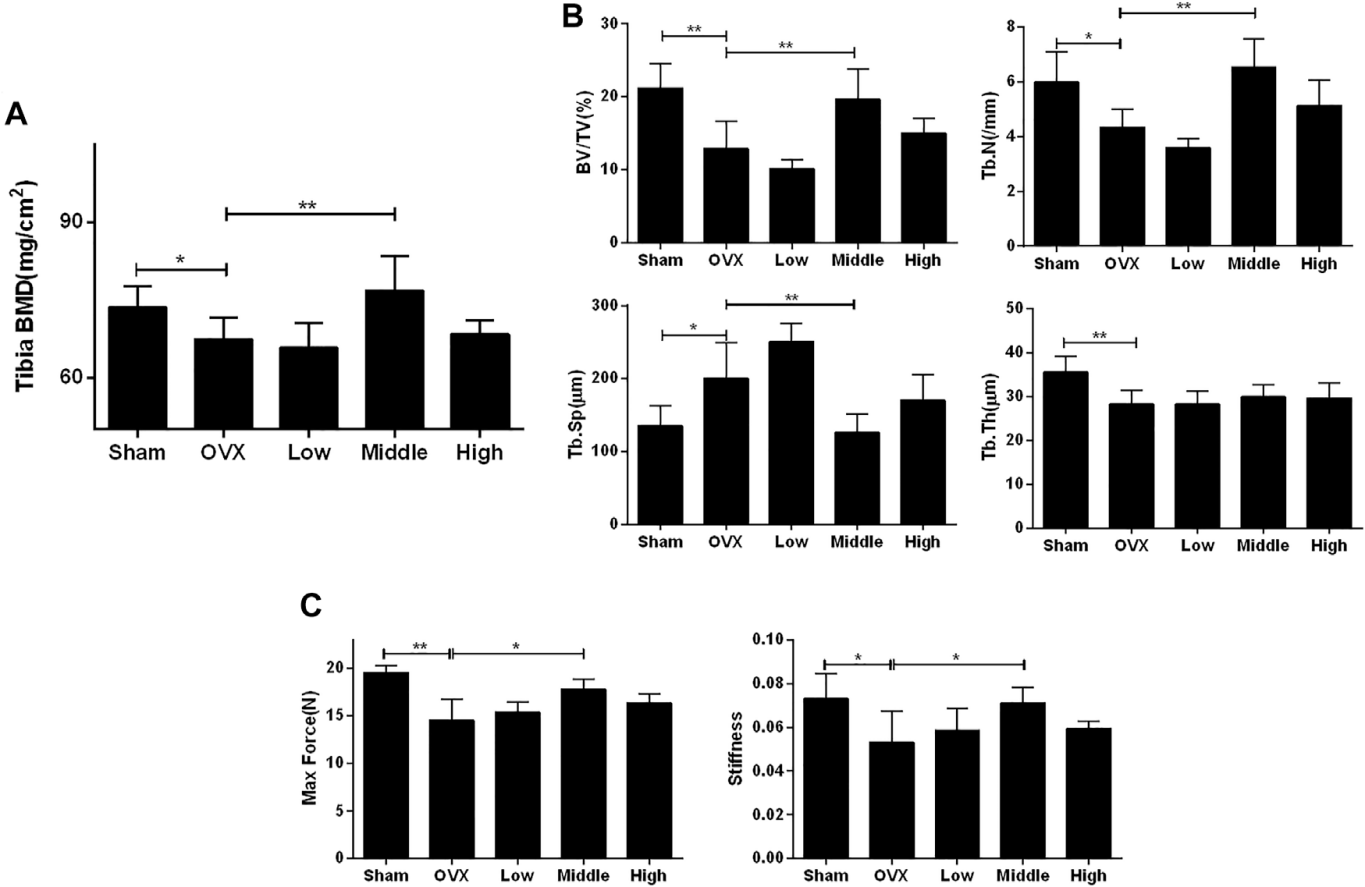
Nitazoxanide prevented OVX-induced bone loss and bone stiffness decrease. **(A)** The left whole tibia BMD were measured. *n* = 10–13. **(B)** The tibias of mice were scanned by high-resolution micro-CT. Calculation of the microarchitectural parameters was performed. *n* = 6. **(C)** The maximum load and stiffness were determined in a three-point bending test. *n* = 6. Data are shown as mean ± SD. BMD, bone mineral density; BV/TV, bone volume/tissue volume; Tb.N, trabecular number; Tb.Sp, trabecular separation; Tb.Th, trabecular thickness. *p value <0.05, **p value <0.01.

**FIGURE 4:**
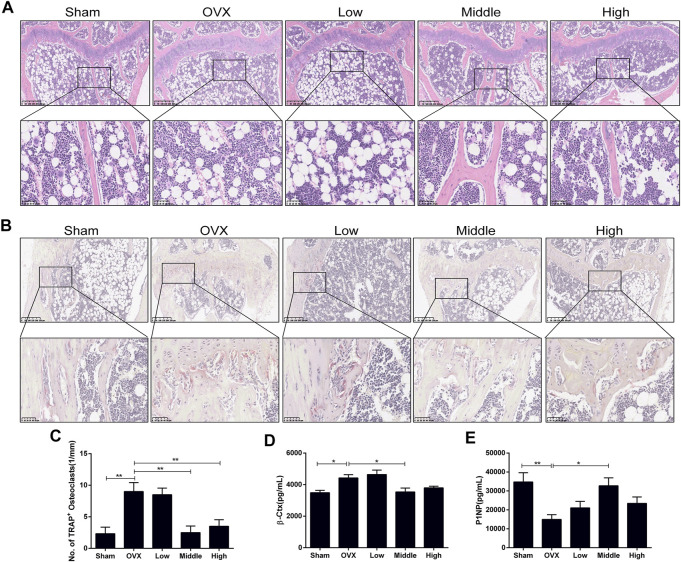
Nitazoxanide protected against OVX-induced trabecular bone loss and osteoclasts formation in vivo. **(A)** Tibia sections were stained with H&E. *n* = 6. **(B)** Sections of tibias were stained with TRAP. *n* = 6. **(C)** The number of multinucleated osteoclasts was counted. **(D–E)** Serum β-Ctx and P1NP were detected by ELISA. *n* = 8. Data are shown as mean ± SD. OVX = ovariectomy; TRAP = tartrate-resistant acid phosphatase; β-Ctx = beta-isomerized C-terminal telopeptides; P1NP = procollagen 1 N-terminal propeptide. *p value <0.05, **p value <0.01.

An ovariectomized mouse model was utilized to validate the anti-osteoporosis activity of NTZ. As shown in Supplementary Figure S2, increased mouse body weight and decreased uterus weight were confirmed. CMC or different doses of NTZ were administered intragastrically into OVX mice once daily for 12 weeks. Bone mineral density (BMD) analyses were performed to evaluate the effect of NTZ on bone quantity in the entire femur and tibia. Compared with the sham group, there was a significant decrease in the BMD in the OVX group (Figure 3A; Supplementary Figure S3A). The middle-dose NTZ (100 mg/kg/d) treatment prevented BMD reduction in tibia (Figure 3A), whereas the effect was not significant in femur (Supplementary Figure S3A). Micro-computed tomography provided the changes of microstructural parameters. In tibia, the BV/TV, Tb.N and Tb.Th displayed a strong decrease and Tb.Sp increase in the OVX group, compared with those of the sham group (Figure 3B). The middle-dose NTZ treatment reversed the changes of microstructural parameters due to OVX except Tb.Th (Figure 3B). Consistent with the BMD results, microstructural parameters of the distal femur in NTZ treated group did not show any significant difference compared with those of the OVX group (Supplementary Figure S3B). Three-point bending tests were used to evaluate the mechanical integrity of tibia. As shown in Figure 3C, the middle-dose NTZ treatment group demonstrated significantly higher bone stiffness than that in the OVX group. The trend for a beneficial effect of middle-dose NTZ on the bone mass of OVX mice was further verified through histologic assessments (H&E staining). The trabecular bone in the OVX group was significantly reduced compared with the sham group, while the NTZ treatment group preserved trabecular bone, especially in the middle and high dose NTZ treated groups (Figure 4A). TRAP staining images, the number of osteoclasts and the levels of serum markers (P1NP and β-Ctx) also confirmed the protective effect of middle-dose NTZ on bone quantity in the OVX model (Figures 4B–E). All these *in vivo* results were similar to the inhibitory effect of NTZ on osteoclastogenesis *in vitro*.

### NTZ Inhibits RANKL-Induced Activation of the STAT3 Pathway and Reduces Ca^2+^ Fluorescence Intensity

**FIGURE 5:**
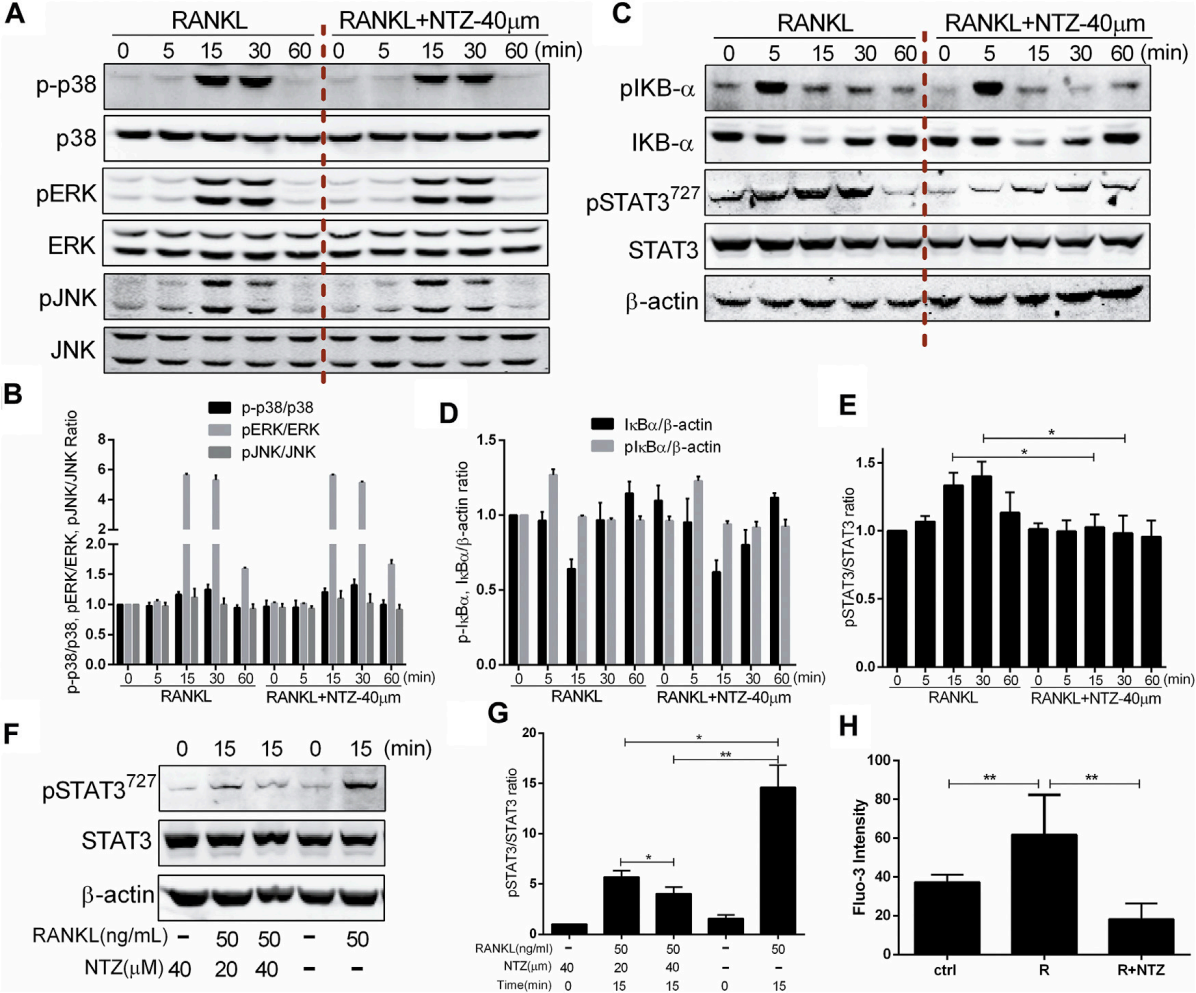
Nitazoxanide impaired RANKL-induced STAT3 phosphorylation and reduced Ca2+ fluorescence intensity. **(A)** RAW264.7 cells were treated with or without 40 µM NTZ for 2 h and then treated with 50 ng/ml RANKL for the indicated periods. Cell lysates were analyzed using immunoblotting. The expression of phosphorylated p38, ERK and JNK was evaluated. Total JNK, p38 and ERK levels were used as loading controls. **(B)** The relative ratios of p-p38, pERK and pJNK to their non-phosphorylated forms were quantified by ImageJ. **(C)** Cells were treated as in **(A)**, cell lysates were analyzed using western blotting. Expression of pIκBα, IκBα, pSTAT3 and STAT3 were evaluated. β-actin was used as a loading control. **(D)** The quantitative analysis of pIκBα and IκBα expression between the NTZ-treated group and control group. **(E)** The quantitative analysis of pSTAT3/STAT3 expression between the NTZ-treated group and control group. **(F)** RAW264.7 cells were pretreated with NTZ for 2 h, and RANKL was subsequently added for 15 min pSTAT3 and STAT3 were detected. β-actin was used as a loading control. **(G)** The relative ratios of pSTAT3 to STAT3 were quantified by ImageJ. **(H)** RAW264.7 cells were treated with 50 ng/ml RANKL, with or without 40 µM NTZ for 48 h. Fluo-3 intensity was analyzed using immunofluorescence. The mean change in calcium intensity per cell in each group. Error bars represent mean ± SD from three independent experiments. ctrl, control; NTZ, nitazoxanide; R, RANKL. *p value <0.05, **p value <0.01.

Osteoclast differentiation is inseparable from RANKL-induced activation of the MAPK, NF-κB, STAT3 pathways and Ca^2+^ oscillation. The underlying mechanisms, by which NTZ inhibited osteoclast formation and bone resorption, were clarified through detecting the phosphorylation of a variety of proteins at different time points. In the MAPK pathway, ERK1/2, P38, and JNK phosphorylation were investigated, and the results showed that the phosphorylation of these proteins was not altered by NTZ treatment (Figures 5A,B). A similar result was observed in the phosphorylation and degradation of IκB-α, suggesting that NTZ does not interfere with the RANKL-mediated IκB-α-dependent NF-κB activation signal (Figures 5C,D). By contrast, NTZ significantly inhibited RANKL-induced activation of the STAT3 pathway by disrupting STAT3 phosphorylation (Figures 5C,E). Moreover, the inhibitory effect of NTZ on STAT3 was concentration dependent (Figures 5F,G). NTZ also significantly reduced RANKL-induced Ca^2+^ fluorescence intensity in RAW264.7 (Figure 5H), which suggested that abnormal calcium oscillation partially contributed to the impaired osteoclastogenesis.

### NTZ Suppressed RANKL-Induced Expression of Transcription Factor NFATc1

**FIGURE 6:**
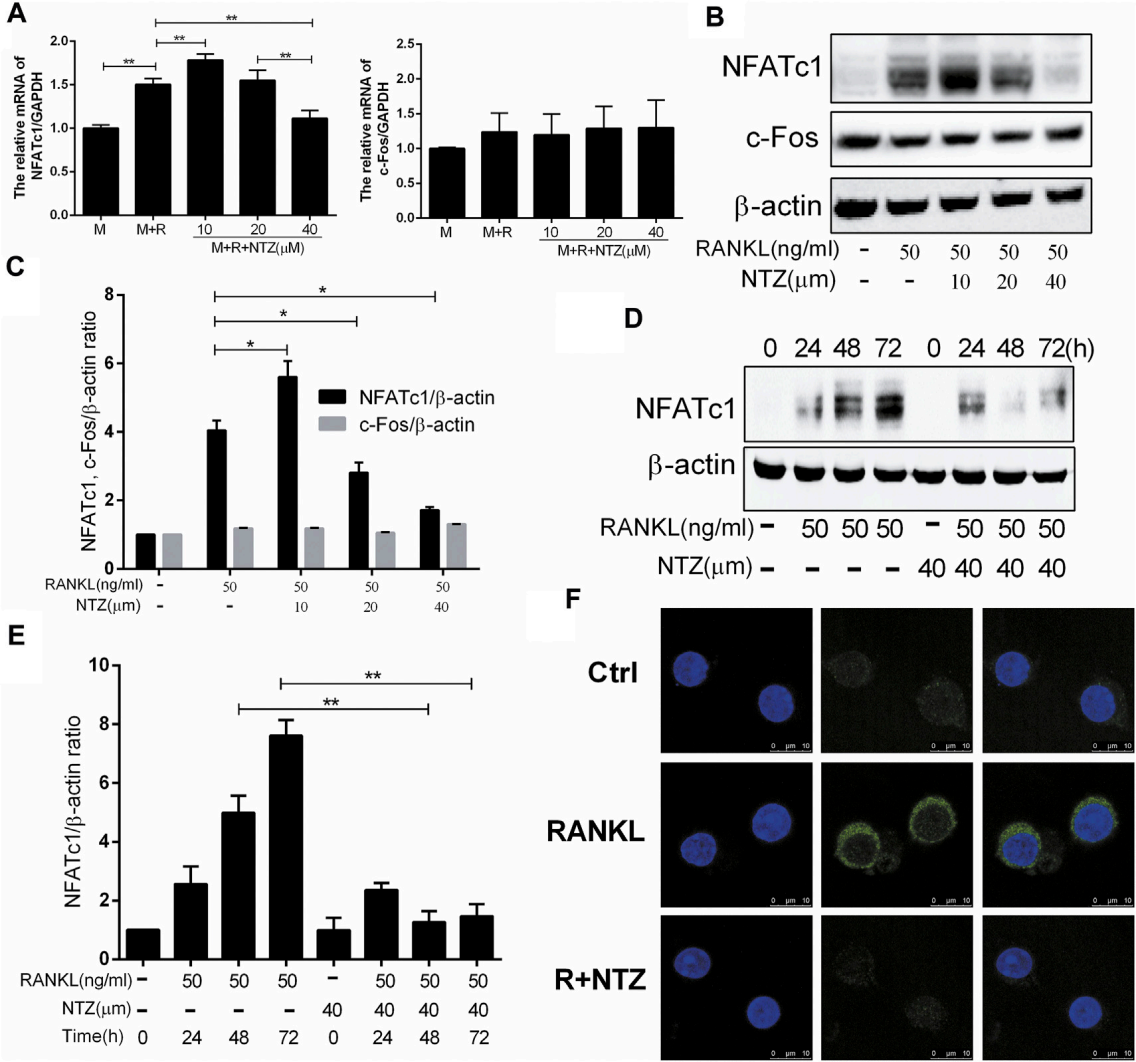
Nitazoxanide suppressed RANKL-induced NFATc1 expression. **(A)** BMMs were treated with various concentrations of NTZ, M-CSF (10 ng/ml), and RANKL (50 ng/ml) for 3 days. NFATc1 and c-Fos expression was determined by real-time PCR. **(B)** RAW264.7 cells were pretreated with the indicated NTZ doses for 2 h, and RANKL was subsequently added for 3 days. Expression of NFATc1 and c-Fos was detected by western blotting. β-actin was used as a loading control. **(C)** The relative ratios of NFATc1 and c-Fos to β-actin were quantified by ImageJ. **(D)** RAW264.7 cells were pretreated with 40 µM NTZ for 2 h, then RANKL was added for the indicated time. Cell lysates was used to analyzed the expression of NFATc1. β-actin was used as a loading control. **(E)** The quantitative analysis of NFATc1/β-actin expression was performed by ImageJ **(F)** Cells were treated as in **(B)**, NFATc1 expression was analyzed using immunofluorescence. Error bars represent mean ± SD from three independent experiments. ctrl = control; M = M-CSF; NTZ = nitazoxanide; R = RANKL. *p value <0.05, **p value <0.01.

NFATc1 and c-Fos are the most important transcription regulators that play a key role in RANKL-induced osteoclastogenesis, and promote osteoclast-specific genes expression. The effects of NTZ on NFATc1 and c-Fos expression were evaluated. 40 µM NTZ significantly inhibited NFATc1 expression at the mRNA and protein levels, but had little effect on the expression of c-Fos (Figures 6A–C). Differently, 10 µM NTZ increased NFATc1 expression (Figures 6A–C). Although 20 µM NTZ did not reduce NFATc1 mRNA expression, it indeed inhibited NFATc1 expression at the protein level (Figures 6A–C). These results coincided with the aforementioned effect of NTZ on osteoclastogenesis. Additionally, RANKL upregulated NFATc1 expression in a time-dependent manner, while NTZ significantly inhibited the expression of NFATc1 after RANKL stimulation for 48 and 72 h (Figures 6D,E). The cellular immunofluorescence experiment indicated that NTZ significantly inhibited cytoplasmic NFATc1 expression (Figure 6F). These results suggested that NTZ effectively suppressed RANKL-induced osteoclastogenesis through downregulating the expression of NFATc1, but not that of c-Fos.

### NTZ Impaired the Activity of STAT3 on NFATc1 Transcription

**FIGURE 7:**
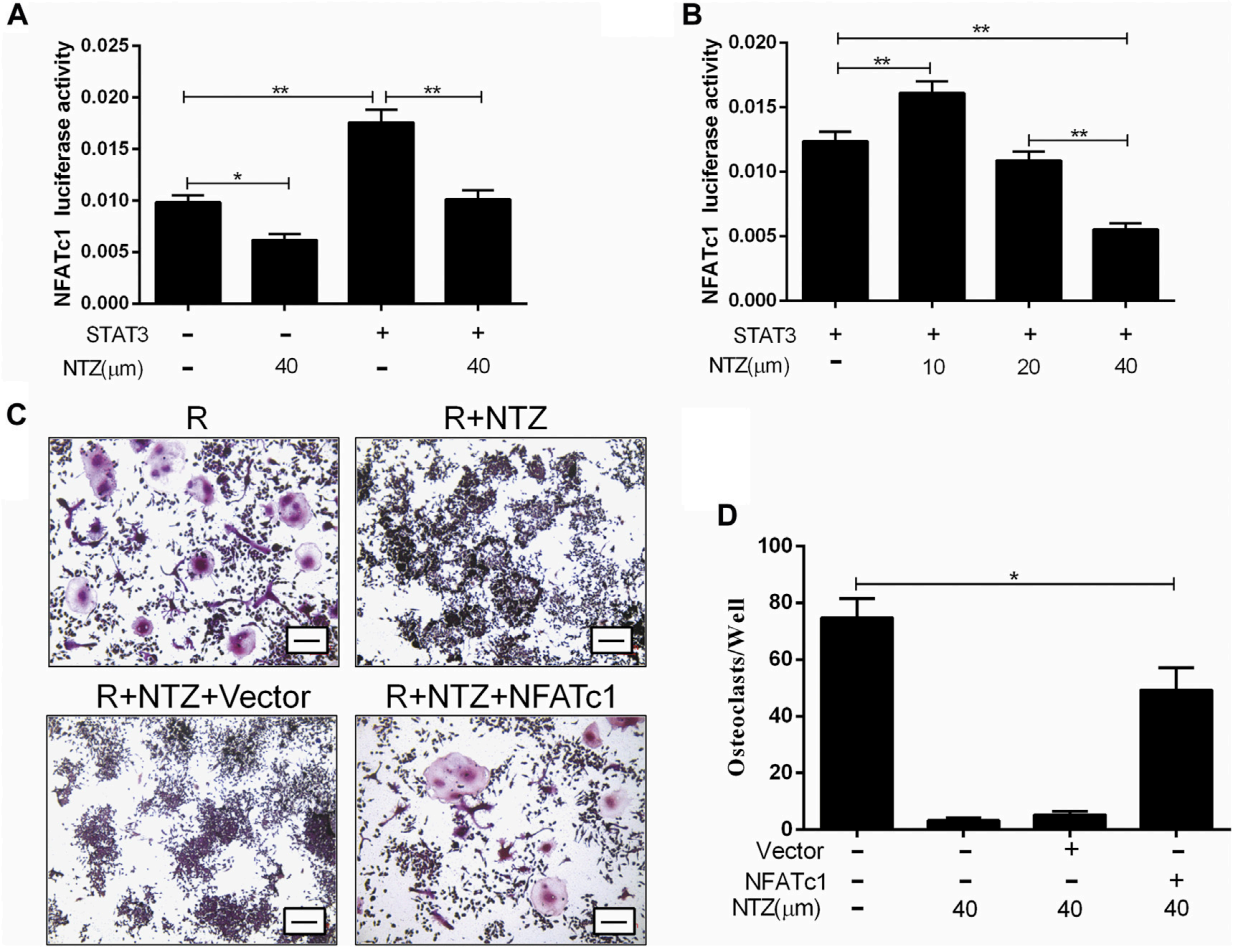
Nitazoxanide impaired STAT3 activity on NFATc1 transcription. **(A, B)** RAW264.7 cells were transfected with or without STAT3, together with NFATc1-Luc, and were subsequently treated with 40 µM NTZ **(A)** or a range of NTZ concentrations **(B)**. NFATc1 luciferase activity was analyzed. **(C)** RAW264.7 cells were transfected with NAFTc1 or control plasmid, and then were stimulated with RANKL (50 ng/ml) in the presence of 40 µM NTZ for 7 days. TRAP staining was performed at day 7. Scale bar = 100 µm. **(D)** The number of TRAP^+^ osteoclasts was counted under light microscopy. Error bars represent mean ± SD from three independent experiments. NTZ = nitazoxanide; R = RANKL. *p value <0.05, **p value <0.01.

The STAT3-NFATc1 signaling is another supplement to the RANKL classic signaling pathway, which is involved in the differentiation and maturation of osteoclasts (Yang et al., 2019). Therefore, we investigated the effect of NTZ on the interaction between STAT3 and the NFATc1 promoter. Constitutively active STAT3 promoted NFATc1 promoter activity more strongly than endogenic STAT3, and 40 µM NTZ treatment significantly reduced NFATc1 promoter activity (Figures 7A,B). Conversely, 10 µM NTZ increased NFATc1 promoter activity, suggesting that the inhibitory effect of NTZ on NFATc1 promoter activity was concentration-dependent (Figure 7B). In addition, enforced expression of NFATc1 in RAW264.7 cells partly rescued the impaired osteoclast differentiation in the NTZ treated group (Figures 7C,D). All these results imply that STAT3–NFATc1 signaling is a potential pharmacological target for NTZ to inhibit RANKL-induced osteoclastogenesis.

## Discussion

The structure and integrity of bone is inseparable from the precise bone remodeling, which is a balance between anabolism and catabolism. Osteoclasts and osteoblasts are essential cell types in these processes, and they interact with each other through complex cross-talk (Chen et al., 2018). When the bone resorption activity of osteoclasts exceeds the bone formation of osteoblasts, it will cause skeletal diseases, such as OP, rheumatoid arthritis and periodontal diseases (Hienz et al., 2015; Tateiwa et al., 2019). Therapeutic regimens targeting excessive osteoclasts activation have been developed, especially the interference strategies for the downstream signaling pathways of RANKL (Xie et al., 2018; Hsu et al., 2019; Zhao et al., 2019). However, because few compounds have advanced to early-phase clinical trials (Zhou et al., 2020), the development of safe and effective therapies is still on the way. A number of studies in recent years have shown that STAT3 is critical in bone catabolism (Cheon et al., 2016; Li et al., 2018; Yang et al., 2019), which suggests that STAT3 can be used as a potential pharmacological molecular target for the development of anti-bone metabolic disease drugs.

Traditional drug discovery and development is a time-consuming, laborious and cost-intensive process. Unfortunately, there is an ever widening productivity gap between the increasing research and development investment and the declining number of new drugs approved by the FDA (Boguski et al., 2009). For this reason, there has been a shift of R&D towards more predictable, reliable and cost-effective drug development strategies, such as drug repurposing (Parvathaneni et al., 2019). NTZ (Alinia^®^) is an FDA approved antiprotozoal, safe, inexpensive and well-tolerated established drug (Fox and Saravolatz, 2005), which has been proven to have a wide range of biological effects (Shakya et al., 2018). The most recent studies suggested that NTZ may be used as a novel STAT3 pathway inhibitor for the treatment of neoplastic diseases (Tantawy et al., 2020; Lu et al., 2021). Given the critical role of the STAT3 signaling pathway in osteoclastogenesis, the present study proposed that NTZ has the great potential for repurposing against OP.

This study demonstrated that NTZ could inhibit osteoclast formation, upregulation of osteoclast-specific marker proteins and bone resorption induced by RANKL in a dose-dependent manner. NTZ displayed only slight inhibition of BMM proliferation, with complete elimination of osteoclastogenesis elicited by this compound occurs at 40 µM. Most importantly, NTZ directly targets the differentiation of osteoclasts from their precursors at the early stage, as demonstrated by delayed NTZ treatment failing to completely inhibit osteoclast formation. Continuous NTZ treatment still effectively inhibited the bone resorption, although this effect was mostly correlated with the reduced number of mature osteoclasts. These data imply that NTZ not only plays an inhibitory role at the early stage of RANKL-induced osteoclastogenesis, but also exerts an inhibitory effect at the following stage of bone resorption, suggesting continuous use of NTZ instead of early discontinuation may be beneficial for the treatment of OP. This proposition coincides with the classic anti-osteoporosis treatment strategy of bisphosphonates (Black et al., 2012). Subsequently, an OVX-induced estrogen deficiency mouse model was used to determine the effect of NTZ on bone loss *in vivo*. NTZ administration protected against OVX-induced tibia bone loss as characterized by BMD preservation, bone strength increase, improved microstructural parameters and osteoclast number reduction. Although the changing trend of BMD and microstructural parameters in femur was similar to that of tibia, statistical differences were not observed. The reason may be related to the difference between the tibia and femur, such as different effective dosage and duration of NTZ treatment. Other studies also found that femurs and tibias responses differently under estrogen withdrawal or alendronate treatment(Thompson et al., 1992; Klinck and Boyd, 2008; Zhao et al., 2018). Furthermore, only the middle dose group showed a positive effect in in vivo, which also prompted further studies to explore a more suitable dose and the potential to promote osteoblast in addition to its anti-bone resorption effect of NTZ in the future.

**FIGURE 8:**
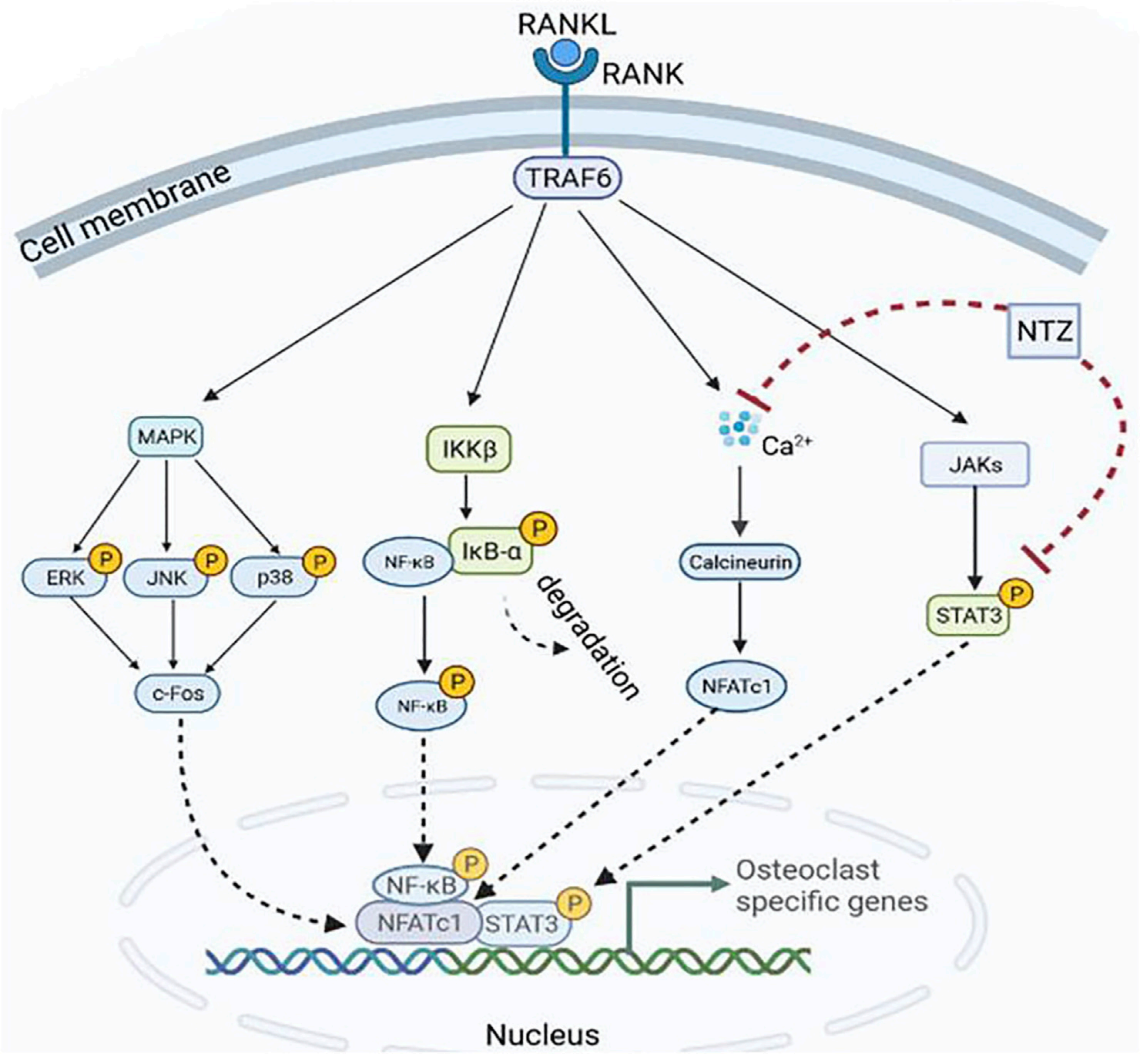
A schematic diagram showing the proposed inhibitory mechanism of Nitazoxanide in inhibiting RANKL-induced osteoclastogenesis.

Mechanistic assessment of the drug effect in preclinical models is one of the key steps for the drug repurposing strategy (Pushpakom et al., 2019). The present determined the effect of NTZ on the RANKL-activated signaling pathways. Interestingly, NTZ significantly attenuated RANKL-induced STAT3 pathway activation, but not that of canonical MAPK or the IκB-α-dependent NF-κB signal. When considering the proliferative effects of MAPK and NF-κB activation (Lee et al., 2002; Park et al., 2017), this result likely explains the limited effect of the indicated NTZ dose on the proliferation of osteoclast precursors. By contrast, NTZ significantly inhibited the expression of NFATc1, which is a master transcriptional regulator of osteoclast differentiation and formation (Takayanagi et al., 2002; Aliprantis et al., 2008), suggesting that the reduction in NFATc1 expression was not achieved by disrupting NF-κB signaling. Coincidentally, research by Yang et al. (2019) found that STAT3 was a regulator for the transcription of NFATc1 signaling during the process of osteoclast differentiation. It is reasonable to consider that NTZ might affect NFATc1 transcriptional activity by interfering with STAT3 signaling. According to the luciferase reporter results, NTZ was able to attenuate NFATc1 transcription activity in accordance with its inhibitory effect on osteoclast formation. Furthermore, overexpression of NFATc1 in NTZ-treated RAW264.7 cells apparently rescued osteoclast formation, as demonstrated by the increased number of TRAP positive osteoclasts. Additionally, the reduction of Ca^2+^ signaling intensity by NTZ may have partially contributed to the attenuation of NFATc1 activity. Thus, STAT3-Ca^2+^-NFATc1 signaling axis may be the potential molecular target of NTZ during RANKL-induced osteoclastogenesis (Figure 8).

Another important issue worth discussing is the dose-dependent efficacy of NTZ. In the present study, it was found that low-dose NTZ (10 µM) promoted, rather than blunted, osteoclastogenesis *in vitro*, characterized by the increased osteoclast number and osteoclastic marker genes expression. Although 10 µM NTZ *in vitro* experiment cannot be completely equated with 50 mg/kg/d *in vivo* experiment, low-dose NTZ (50 mg/kg/d *in vivo*) exhibited a similar trend in the OVX model to promote, rather than protect against, bone loss. Moreover, only the middle-dose NTZ (100 mg/kg/d *in vivo*) was obviously effective in preventing OVX-induced bone loss. This suggests a limited therapeutic range for NTZ with current formulation in terms of its osteoprotective effect. Therefore, it should be clearly realized that this potential benefit of NTZ on bone metabolism may be limited to a small therapeutic window. However, this limitation should not completely obliterate the potential future of NTZ. Further optimizations will be necessary to modify this well-established drug to broaden its therapeutic window. In addition, the low aqueous solubility and bioavailability of NTZ has already been noticed in the treatment of cancer and infectious diseases and many deviations on its structure is currently ongoing to solve this problem (Wang et al., 2018; Lokhande and Devarajan, 2021).

In summary, this study first discovered that NTZ is able to inhibit differentiation of BMMs into osteoclasts and bone resorption activity of mature osteoclasts. These inhibitory effects of NTZ occur through suppression of STAT3 phosphorylation and reduction of the Ca^2+^ fluorescence intensity followed by down-regulated NFATc1 expression. These results suggest that NTZ could be a potential agent in the treatment of osteoclast-related diseases.

## Data Availability

The original contributions presented in the study are included in the article/Supplementary Material, further inquiries can be directed to the corresponding authors.
