# Corrigendum: Nitazoxanide, an antiprotozoal drug, reduces bone loss in ovariectomized mice by inhibition of RANKL-induced osteoclastogenesis

**DOI:** 10.3389/fphar.2023.1247393

**Published:** 2023-07-26

**Authors:** Chang-hong Li, Zi-rui Lü, Zhen-da Zhao, Xin-yu Wang, Hui-jie Leng, Yan Niu, Mo-pei Wang

**Affiliations:** ^1^ Department of Rheumatology and Immunology, Peking University Third Hospital, Beijing, China; ^2^ Osteoporosis and Bone Metabolic Diseases Center, Peking University Third Hospital, Beijing, China; ^3^ Department of Medicinal Chemistry, School of Pharmaceutical Sciences, Peking University Health Science Center, Beijing, China; ^4^ Department of Orthopaedics, Peking University Third Hospital, Beijing, China; ^5^ Beijing Key Laboratory of Spinal Disease Research, Beijing, China; ^6^ Department of Tumor Chemotherapy and Radiation Sickness, Peking University Third Hospital, Beijing, China

**Keywords:** nitazoxanide, stat3, osteoclastogenesis, Nfatc1, osteoporosis

In the published article, there was an error in the **Funding** statement. The original statement read: “This work was supported by National Natural Science Foundation of China (No. 81501387, No. 12172011, No. 11872076), Bethune Osteoporosis Foundation Project (G-X-2020-1107-02) and Youth Backbone Incubation Fund of Peking University Third Hospital (BYSYFY2021027).” This statement should be corrected as follows:

## Funding

This work was supported by Scientific Research Foundation of Returned Scholars of Peking University Third Hospital (LXHG2018003), National Natural Science Foundation of China (Nos 81501387, 12172011, and 11872076), Bethune Osteoporosis Foundation Project (G-X-2020-1107-02), and Youth Backbone Incubation Fund of Peking University Third Hospital (BYSYFY2021027).

The authors apologize for this error and state that this does not change the scientific conclusions of the article in any way. The original article has been updated.

