# Supplementary material for: Nitazoxanide, an Antiprotozoal Drug, Reduces Bone Loss in Ovariectomized Mice by Inhibition of RANKL-Induced Osteoclastogenesis
Source: Front Pharmacol. 2021 Dec 9;12:781640. doi: 10.3389/fphar.2021.781640 (PMC8696474; doi:10.3389/fphar.2021.781640)
Supplement: Supplementary file 1 [file Table1.docx]

**Supplementary Table S1 The primer sequences used in the RT-PCR assays.**

| Genes | Forward | Reverse |
| --- | --- | --- |
| *GAPDH* | AATGGATTTGGACGCATTGGT | TTTGCACTGGTACGTGTTGAT |
| *TRAP* | CCAATGCCAAAGAGATCGC | TCTGTGCAG AGACGTTGCCAAG |
| *MMP9* | CTGGACAGCCAGACACTAAAG | CTCGCGGC AAGTCTTCAGAG |
| *cathepsin-K* | GTTGT ATGTATAACGCCACGGC | CTTTCTCGTTCCCCACAGGA |
| *c-Fos* | CGCAGAGCATCGGCAGAAGG | TCTTGCAGGCAGGTCGGTGG |
| *NFATc1* | CCGTTGCTTCCAGAAAATAACA | TGTGGGATGTGAACTCGGAA |
