# Supplementary material for: Nitazoxanide, an Antiprotozoal Drug, Reduces Bone Loss in Ovariectomized Mice by Inhibition of RANKL-Induced Osteoclastogenesis
Source: Front Pharmacol. 2021 Dec 9;12:781640. doi: 10.3389/fphar.2021.781640 (PMC8696474; doi:10.3389/fphar.2021.781640)
Supplement: Supplementary file 5 [file DataSheet1.docx]

**Supplementary Figure S1.** Chemical structures of Nitazoxanide.

**Supplementary Figure S2. Validation of Ovariectomized (OVX) mouse model.** A, Uterus was isolated and weighted after mice were sacrificed. B, The body weight of mice was routinely recorded during the experiment. n = 10-13. Data are shown as mean ± SD. Values of body weight were compared between Sham group and OVX group at 14 week. OVX = ovariectomy. **p* value ＜0.05, ***p* value ＜0.01.

**Supplementary Figure S3. Nitazoxanide has no effect on OVX-induced femoral bone loss.** (A) The left whole-femur BMD were measured. n = 10-13. (B) The femurs of mice were scanned with a high-resolution micro-CT. Calculation of the microarchitectural parameters was performed. n = 6. Data are shown as mean ± S. BMD = bone mineral density; BV/TV = bone volume/tissue volume; Tb.N = trabecular number; Tb.Sp = trabecular separation; Tb.Th = trabecular thickness; OVX = ovariectomy. **p* value ＜0.05.
